# Antimicrobial activity of ginsenoside Rh2 against *Enterococcus faecalis* and its biofilm

**DOI:** 10.3389/fmicb.2026.1979316

**Published:** 2026-10-09

**Authors:** Chaoran Liu, Yanchun Wang, Shu Zhou, Guanghong Han, Yingxian Zhai

**Affiliations:** 1 Hospital of Stomatology, Jilin University, Changchun, Jilin, China; 2 Department of Anesthesiology, Jilin Cancer Hospital, Changchun, Jilin, China; 3 Key Laboratory of Pathobiology, Ministry of Education, Jilin University, Changchun, China

**Keywords:** 20(S)-ginsenoside Rh2, antibacterial, bacterial biofilm, *Enterococcus faecalis*, Ginsenoside Rh2

## Abstract

**Background:**

*Enterococcus faecalis* (*E. faecalis*) is frequently associated with persistent root canal infections and treatment failure, and effective strategies for its elimination remain limited. Plant-derived compounds may provide alternative antimicrobial strategies for endodontic treatment.

**Methods:**

In the present study, we evaluated the antimicrobial activity of 20(S)-ginsenoside Rh2 (Rh2), a bioactive compound derived from ginseng, against planktonic and biofilm forms of *E. faecalis*. The effects of Rh2 on extracellular polysaccharide (EPS)-associated carbohydrate content and biofilm metabolic activity were evaluated using the phenol-sulfuric acid and MTT assays, respectively. Biofilm structural alterations were examined by scanning electron microscopy (SEM) and confocal laser scanning microscopy (CLSM). The cytotoxicity of Rh2 toward L929 cells was assessed using an MTT assay, with sodium hypochlorite (*NaClO*) serving as the reference antimicrobial agent.

**Results:**

Rh2 exhibited antibacterial activity against planktonic *E. faecalis*, with a minimum inhibitory concentration (MIC) of 3.125 μg/mL and a minimum bactericidal concentration (MBC) of 6.250 μg/mL. Rh2 inhibited biofilm formation and reduced the biomass of established biofilms at 2 × MIC and 4 × MIC. Rh2 also markedly reduced EPS-associated carbohydrate content, altered biofilm architecture, and decreased the metabolic activity of biofilm-associated bacteria. Under the tested conditions, Rh2 exhibited favorable cellular compatibility at concentrations with antibacterial activity against *E. faecalis*.

**Conclusion:**

These findings demonstrate the *in vitro* antibacterial and antibiofilm activities of Rh2 against *E. faecalis* and support further investigation of its potential as an intracanal antimicrobial agent.

## 1 Introduction

*Enterococcus faecalis (E. faecalis)* is an important pathogen associated with pulpitis, infection-related pulp necrosis, and persistent periapical infection. There is a high detection rate of *E. faecalis* in teeth with persistent or previously treated root canal infections (Sun et al., 2022; Xu et al., 2025). Consequently, to avoid subsequent root canal infections, it is imperative to completely eradicate the drug-resistant bacteria from the root canal system (Ali and Neelakantan, 2022). Because bacteria may persist within the root canal system and dentinal tubules after instrumentation, chemomechanical preparation alone may not be sufficient for complete microbial eradication (Carvalho et al., 2020; Gulabivala and Ng, 2023). Therefore, intracanal irrigants and antimicrobial medicaments are commonly used to enhance root canal disinfection.

However, their use may be limited by incomplete antibacterial efficacy and irritation of periapical tissues. Sodium hypochlorite (*NaClO*) is used to control root canal infections; however, its cytotoxicity may compromise the viability of surrounding tissues (Dede et al., 2023; Sreelekshmi et al., 2026). Chlorhexidine (CHX) has also been investigated for the elimination of *E. faecalis*, but its efficacy may be reduced against bacteria located deep within biofilms, and repeated antimicrobial exposure may contribute to phenotypic adaptation (Wang et al., 2017; Schwarz et al., 2021). Calcium hydroxide [Ca(OH)₂], a commonly used intracanal medicament, exerts antimicrobial activity primarily through hydroxide ions, which can disrupt bacterial membranes, proteins, enzymes, and nucleic acids (Jara et al., 2020). However, *E. faecalis* can tolerate alkaline stress through mechanisms including proton-pump-mediated pH homeostasis, thereby reducing its susceptibility to Ca(OH)₂ (Divakar et al., 2020). These limitations highlight the need for alternative antimicrobial strategies with effective antibacterial activity and favorable biocompatibility.

Increasing attention has therefore been directed toward plant-derived compounds as potential antimicrobial agents for the management of root canal infections. *Panax ginseng* has been reported to possess a broad range of biological activities, including antibacterial, anti-inflammatory, and immunomodulatory effects (Wang et al., 2020; Ito and Ito, 2024). More than 100 ginsenosides have been identified and broadly classified into two major structural groups: protopanaxadiol and protopanaxatriol ginsenosides (Xue et al., 2020). Several ginsenosides, including Rh2, Rd., Rg5, Rh4, and Rg3, have been reported to exhibit activity against oral pathogens, such as *Porphyromonas gingivalis*, *Streptococcus mutans*, *Streptococcus haematobium*, and *Fusobacterium nucleatum* (Cao et al., 2019; Xue et al., 2020; Tan et al., 2026).

20(S)-Ginsenoside Rh2 (Rh2) is a bioactive triterpenoid saponin derived from *Panax ginseng*. Previous studies have demonstrated that Rh2 possesses antibacterial and antibiofilm activities against several oral pathogens and may enhance the activity of conventional antibacterial agents. These findings suggest that Rh2 may have potential as a natural antimicrobial compound for oral infections. Therefore, the present study evaluated the antibacterial and antibiofilm activities of 20(S)-ginsenoside Rh2 against *E. faecalis*, using *NaClO* as a reference antimicrobial agent. The cytotoxicity of Rh2 was also evaluated in L929 fibroblasts to provide an initial assessment of its cellular compatibility for potential endodontic applications.

## 2 Materials and methods

### 2.1 Drug preparations

20(S)-ginsenoside Rh2 (purity > 98%) was obtained from Yuanye Biotechnology Co., Ltd. (Shanghai, China; catalog no. B21062). A 10 mg/mL stock solution was prepared in dimethyl sulfoxide (DMSO). The stock solution was sterilized by filtration through a 0.22-μm membrane and stored in the dark until use. Serial twofold dilutions were prepared in BHI medium as required for each experiment.

### 2.2 Bacterial strain and growth conditions

*Enterococcus faecalis* (ATCC 29212) was cultured in Brain Heart Infusion Broth (BHI; Difco, Sparks, MD, USA) overnight at 37 °C under anaerobic conditions with 5% CO_2_ (Thermo, MA, USA). Cultures were grown to the mid-exponential phase and subsequently transferred to fresh BHI. The bacterial concentration was adjusted to 1.0 × 10^8^ CFU/mL based on optical density measurements. For biofilm formation, bacteria were cultured in Tryptic Soy Broth (TSB; Difco, Sparks, MD, USA) supplemented with 0.75% sucrose under anaerobic conditions at 37 °C.

### 2.3 Antibacterial assay

The minimum inhibitory concentration (MIC) and minimum bactericidal concentration (MBC) of Rh2 against *E. faecalis* were determined using a broth microdilution assay according to the Clinical and Laboratory Standards Institute (CLSI) method (Humphries et al., 2018). An *E. faecalis* suspension at 1.0 × 10^8^ CFU/mL was inoculated into freshly prepared BHI at a ratio of 1:100, and 198 μL of the resulting bacterial suspension was transferred to each well of a microplate. Rh2 was serially diluted twofold, and 2 μL of each dilution was added to the corresponding wells to obtain final concentrations ranging from 1.563 to 100 μg/mL and a final bacterial concentration of 1.0 × 10^6^ CFU/mL. The plates were incubated anaerobically at 37 °C for 24 h. *NaClO* was used as a positive control, and 2 μL of DMSO to the blank control. The MIC value of Rh2 was the lowest concentration that produced no visible growth. In order to investigate the MBC value, 100 μL from wells showing no visible growth in the microdilution broth test was inoculated onto BHI solid medium. The MBC was defined as the lowest concentration that resulted in a 99.9% reduction in viable bacteria. Three replicate wells were included for each condition, and all experiments were performed in triplicate.

### 2.4 Growth curves of *Enterococcus faecalis*

The overnight culture was diluted 1:100 in fresh BHI broth, and 198 μL of the bacterial suspension was transferred to each well of a 96-well microplate. Rh2 was serially diluted twofold, and 2 μL of each dilution was added to obtain final concentrations ranging from 0.391 to 3.125 μg/mL. Optical density at 600 nm (OD₆₀₀) was measured every 2 h for 12 h using a microplate reader (Bio-Rad Laboratories, CA, USA), and growth curves were plotted accordingly. *NaClO* was used as the positive control, and DMSO was included as the vehicle control. Three replicate wells were included for each condition, and all experiments were performed in triplicate.

### 2.5 Inhibition of biofilm formation

Biofilm biomass was quantified using a crystal violet (CV) assay in 96-well microplates (He et al., 2019; Wint et al., 2024). The overnight cultures were finally diluted to 1.0 × 10^6^ CFU/mL in TSB medium with 0.75% sucrose and supplemented with Rh2 at different concentrations (the final concentration of Rh2 ranged from 0.391 μg/mL to 3.125 μg/mL). The volumes of bacterial culture medium and reagents are the same as those used in the Antibacterial assay. The control group received TSB medium without any medications. After 48 h of static incubation, the supernatant was removed, and the plates were gently rinsed with 100 μL phosphate-buffered saline (PBS) (Solarbio, Beijing, China) and dried at room temperature. 200 μL of 0.1% crystal violet was used to stain the cells for 20 min at room temperature. The OD_595_ of crystal violet was measured using a microplate reader (Bio-Rad Laboratories, CA, USA) after it had been dissolved in 200 μL of 30% acetic acid. The biofilm formation rate was calculated as follows: Biofilm formation (%) = (N_sample_/ N_blank_) / 100%. The positive control was *NaClO*. An additional 2 μL of DMSO was added to the blank control group. Three replicate samples were placed in each well. All experiments were performed in triplicate.

### 2.6 Effects on established biofilm biomass

Biofilms were allowed to form in 96-well microplates for 48 h under anaerobic conditions with 5% CO₂ at 37 °C. After incubation, biofilm samples were washed three times with PBS and treated with Rh2 at concentrations ranging from 0.391 μg/mL to 12.500 μg/mL in TSB medium containing 0.75% sucrose. The volumes of bacterial culture medium and reagents are the same as those used in the Antibacterial assay. The final bacterial concentration was 1.0 × 10^6^ CFU/mL. After 24 h of incubation, non-adherent bacteria were removed by washing three times with 100 μL PBS. The biofilm samples were then stained with crystal violet, as per the biofilm inhibition assay protocol. Untreated biofilm samples served as controls. Biofilm biomass was quantified by measuring OD₅₉₅ using a microplate reader (Bio-Rad Laboratories, CA, USA). The remaining biofilm rate was calculated as follows: Remaining biofilm (%) = (N_sample_/ N_blank_)/ 100%. *NaClO* served as a positive control. Three replicate samples were placed in each well. All experiments were performed in triplicate.

### 2.7 Extracellular polysaccharides (EPS) measurement

The bacterial suspension was diluted in TSB culture medium containing 0.75% sucrose to the 96-well microplate, and 2 μL of each prepared twofold dilution of Rh2 was added to each well, resulting in a final concentration range of 0.391 μg/mL to 3.125 μg/mL. The final bacterial concentration was 1.0 × 10^6^ CFU/mL in each well. The phenol-sulfuric acid technique was used to investigate the influence of Rh2 on EPS in *E. faecalis* biofilm (Wint et al., 2024). After 48 h of incubation in 5% carbon dioxide at 37 °C, the supernatant was removed, and the plates were gently rinsed with PBS before drying for 20 min at room temperature. Then, 40 μL of sterile distilled water, 40 μL of 6% phenol, and 200 μL of concentrated sulfuric acid were gradually added to each well and incubated at room temperature for 30 min. Finally, microplate reader (Bio-Rad Laboratories, CA, USA) was used to measure OD_492_. A standard curve was plotted using glucose as the standard to determine the concentration of EPS. *NaClO* served as a positive control. Three replicate samples were placed in each well. All experiments were performed in triplicate.

### 2.8 Metabolic activity of biofilm

Biofilm metabolic activity was assessed using the MTT assay (Sigma, USA). The bacterial culture conditions and Rh2 concentrations were the same as those used for EPS analysis, with final Rh2 concentrations ranging from 0 to 3.125 μg/mL and a bacterial concentration of 1.0 × 10^6^ CFU/mL. After 48 h of treatment with Rh2, the culture medium was removed from the culture plates, which were then washed with PBS and left to dry at room temperature. Next, 100 μL of MTT solution (0.5 mg/mL) was added to each well. The plates were incubated for a further 4 h at 37 °C, then removed the MTT solution and dissolved the resulting formazan crystals with 150 μL of 100% DMSO. The OD_490_ was measured using a microplate reader (Bio-Rad Laboratories, CA, USA) after complete dissolution. The metabolic activity rate was calculated as follows: Metabolic activity (%) = (N_sample_/ N_control_) / 100%. *NaClO* was used as the positive control. All experiments were performed in triplicate.

### 2.9 Scanning electron microscopy (SEM)

The effects of Rh2 on the morphology and structure of *E. faecalis* biofilms were examined by SEM (Silva et al., 2020). Cell culture slides were placed in 24-well flat-bottomed polystyrene microplates. Each well was inoculated with 990 μL of an *E. faecalis* suspension at 1.0 × 10^6^ CFU/mL, followed by 10 μL of Rh2 solution to obtain final concentrations ranging from 0.781 to 3.125 μg/mL. After 24 h of static incubation, the supernatant was removed, and the plates were gently washed with PBS before being fixed with a 2.5% glutaraldehyde solution overnight at 4 °C. The biofilms were dried using various ethanol concentrations (30, 40, 50, 60, 70, 80, 90, and 100%) before being completely oven-dried. SEM (Hitachi, Tokyo, Japan) was used to evaluate the samples after they had been coated with gold–palladium. All experiments were performed in triplicate.

### 2.10 Confocal laser scanning microscopy (CLSM)

Biofilms for live/dead cell imaging were generated using the LIVE/DEAD Bac-Light™ Bacterial Viability Kit (Li et al., 2021). The Rh2 concentrations were the same as those used for SEM analysis. After 48 h of static incubation, the supernatant was removed, and the plates were gently washed with PBS. The biofilm was stained with SYTO9 and propidium iodide (PI) in a dark setting, as directed by the Cell Survival and Death Staining Kit instructions. After 30 min, the treated samples were transferred directly to the confocal laser scanning microscopy platform (Olympus, Tokyo, Japan). Images were acquired, and three-dimensional reconstruction and image analysis were performed using Imaris software. Three replicate wells were included for each condition, and all experiments were performed in triplicate.

### 2.11 Cell culture and cytotoxicity assessment

L929 cells (a murine fibroblast cell line) were cultured in Minimum Essential Medium (MEM) with 10% fetal bovine serum and 1% penicillin–streptomycin. Cells at passages 2–3 and at 90% confluence were used in each experiment.

Rh2’s cytotoxicity on L929 was measured using the MTT assay (Silva et al., 2020; Kuang et al., 2021). In a 96-well microtiter plate at 37 °C and 5% CO_2_, L929 cells at a density of 5,000 cells per well were treated with Rh2 at doses ranging from 0 to 25 μg/mL for 24 h. The cells were washed with 1 × PBS and treated in 100 μL of 0.5 mg/mL MTT at 37 °C for 3 *h*. MTT metabolites were dissolved in 150 μL of DMSO and incubated for 10 min at 37 °C in the dark. The absorbance at 450 nm was determined using a microplate reader. Cell viability (%) was calculated as: Viability (%) = (N_sample_/ N_control_)/ 100%. The 50% inhibitory concentration (IC_50_), defined as the concentration required to reduce cell viability by 50%, was estimated from dose–response curves using GraphPad Prism software (San Diego, CA, USA). *NaClO* served as the positive control. Three replicate samples were placed in each well. All experiments were performed in triplicate.

### 2.12 Statistical analysis

Statistical analyses were performed using SPSS version 26.0. Differences between treatment groups and the corresponding control groups were analyzed using Dunnett’s test. Data are presented as the mean ± standard deviation (SD), and *p* < 0.05 was considered statistically significant.

## 3 Results and discussion

### 3.1 Rh2 inhibits the growth of *Enterococcus faecalis*

Rh2 inhibited the growth of *E. faecalis* planktonic cells with an MIC of 3.125 μg/mL and killed planktonic *E. faecalis* at an MBC of 6.250 μg/mL (Table 1). The MBC/MIC ratio of 2 is consistent with bactericidal activity under the conditions tested. Previous studies have also confirmed that Rh2 exhibits antimicrobial activity against oral pathogens, including *E. nucleatum* and *P. gingivalis*, although the MIC values reported were higher than those observed for *E. faecalis* in this study. Such differences should be interpreted cautiously because antimicrobial susceptibility can be influenced by bacterial species and strain, culture conditions, inoculum size, and assay methodology. Under the conditions tested here, *NaClO* exhibited an MIC of 1,800 μg/mL and an MBC of 3,600 μg/mL.

**Table 1:** MICs and MBCs of test agents against *E. faecalis* strains. MIC, minimum inhibitory concentration.

Bacterial strain	MIC (μg/mL)		MBC (μg/mL)	
	Rh2	*NaClO*	Rh2	*NaClO*
*E. faecalis* ATCC 29212	3.125	1800	6.250	3,600

MBC, minimum bactericidal concentration.

Growth-curve analysis further demonstrated the concentration-dependent inhibitory activity of Rh2 against planktonic *E. faecalis*. Rh2 at 1 × MIC and 1/2 × MIC inhibited bacterial growth, whereas 1/4 × MIC and 1/8 × MIC produced no detectable inhibitory effect (Figure 1). This concentration-dependent response suggests that Rh2 may impair bacterial physiological functions before complete growth inhibition or bacterial killing occurs. Previous structure–activity studies have shown that the antimicrobial activity of ginsenosides is associated with their amphiphilic properties and membrane interactions, with less-polar ginsenosides such as Rh2 exhibiting greater membrane affinity. Therefore, membrane disruption and the resulting disruption of membrane homeostasis may be one of the reasons why growth-inhibitory effects are observed at concentrations below the MIC (Xue et al., 2020).

**Figure 1:**
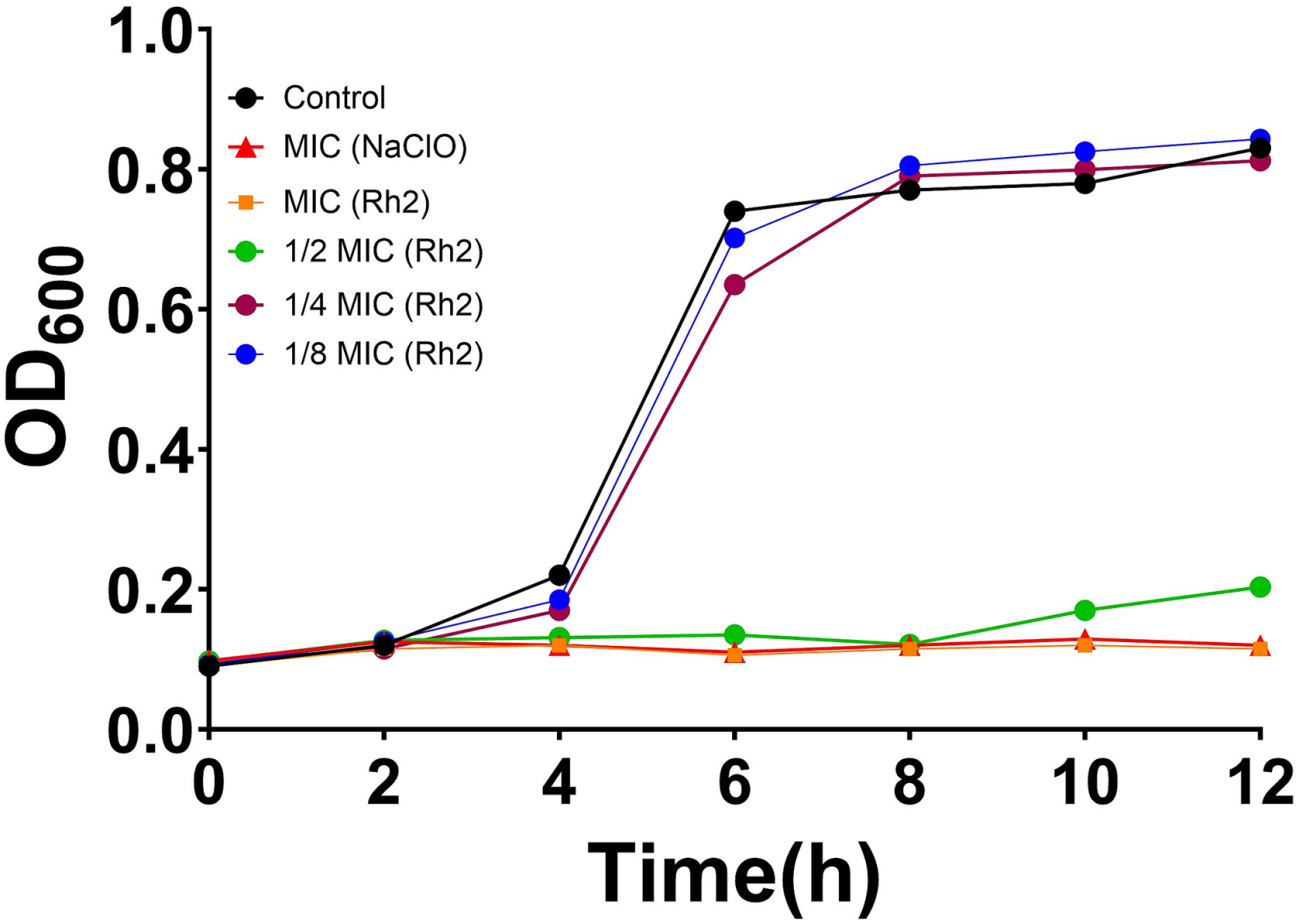
Antibacterial potential of ginsenoside Rh2 against *E. faecalis*. MIC, minimum inhibitory concentration. MBC, minimum bactericidal concentration.

### 3.2 Rh2 inhibits the formation of biofilms and destroys mature biofilms

Biofilm-mediated infection plays an important role in endodontics and periapical disease (Yin et al., 2019; Kreth and Merritt, 2023). Biofilm formation was significantly inhibited by Rh2 in a concentration-dependent manner. Treatment with 1 × MIC and 1/2 × MIC Rh2 reduced *E. faecalis* biofilm formation by 52.18% and 14.56%, respectively, whereas 1/4 × MIC and 1/8 × MIC produced no significant inhibition compared with the control group (Figure 2A). At the MIC, Rh2 produced a greater inhibitory effect on biofilm formation than *NaClO*, which reduced biofilm formation by 38.25% under the same experimental conditions (Figure 2B). For established biofilms, Rh2 at 6.250 and 12.500 μg/mL reduced biofilm biomass by 41.25% and 53.47%, respectively (*p* < 0.05; Figure 3A). The reduction observed at 12.500 μg/mL corresponds to 2 × MBC or 4 × MIC. Under the same conditions, *NaClO* produced a 28.45% reduction at the corresponding concentration (Figure 3B). Rh2 significantly inhibited the formation of *E. faecalis* biofilms; however, higher concentrations were required to substantially reduce existing biofilms. These findings indicate that developing biofilms were more susceptible to Rh2 than established biofilms, which may reflect the protective effect of the mature extracellular matrix on embedded bacterial cells and the associated limitation of antimicrobial penetration. These observations are consistent with previous studies showing that Rh2 can inhibit biofilm biomass accumulation and extracellular polysaccharide production in oral bacteria.

**Figure 2:**
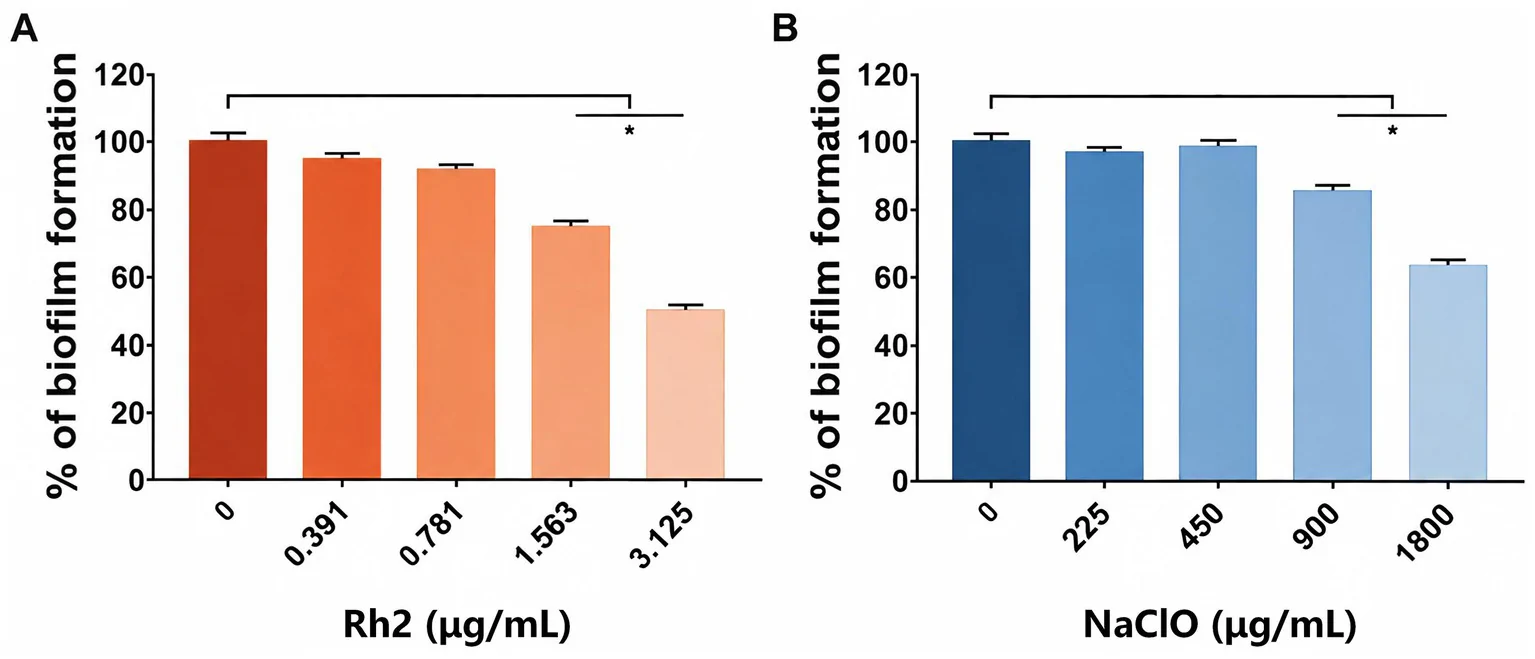
Ginsenoside Rh2 inhibits the formation of *E. faecalis* biofilms. **(A)** Percentage of biofilm formation by different concentrations of Rh2. **(B)** Percentage of biofilm formation by different concentrations of *NaClO*. The blank control group was treated with DMSO. *NaClO* as a positive control. Data are presented as mean ± SD (*n* = 3, with three technical replicates). Statistical analysis was performed using Dunnett’s multiple-comparisons test, *p < 0.05.

**Figure 3:**
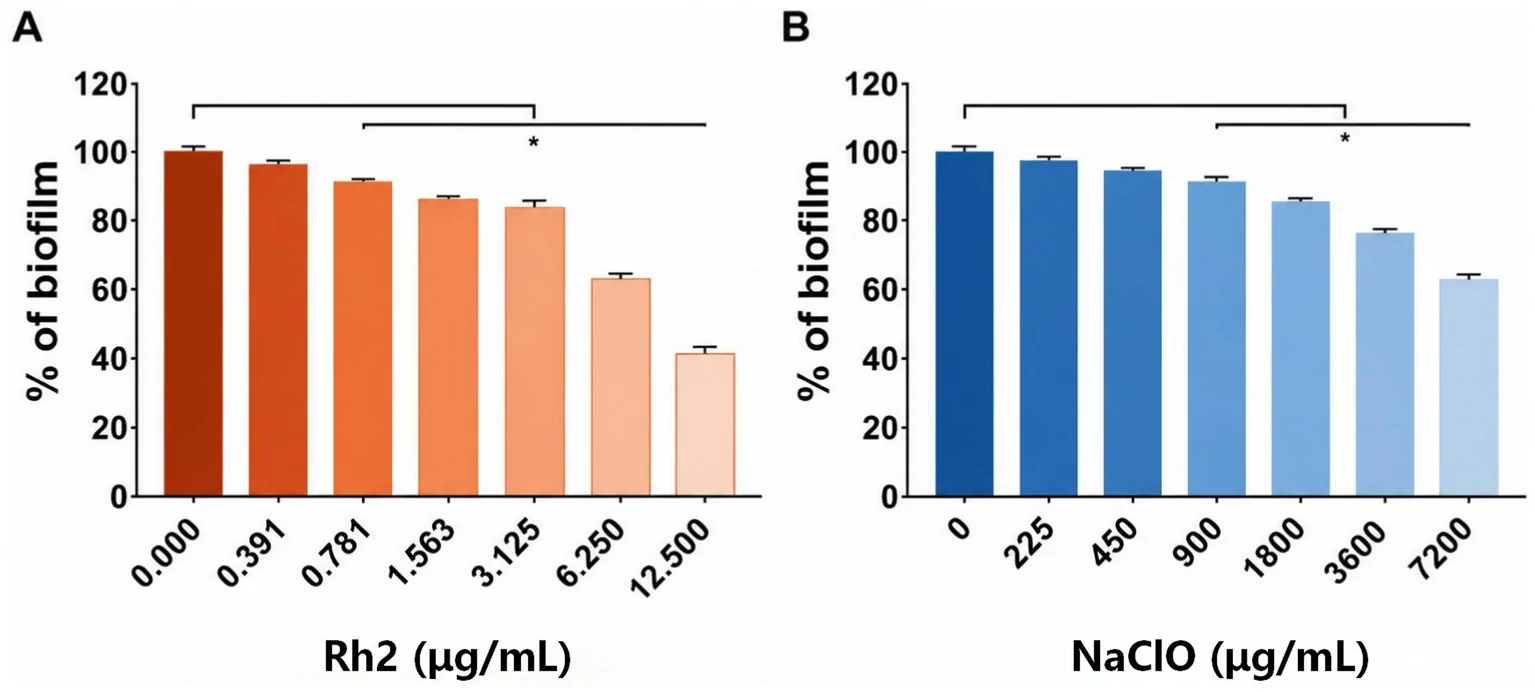
Ginsenoside Rh2 reduces mature *E. faecalis* biofilm biomass. **(A)** Percentage of biofilm remaining after treatment with different concentrations of Rh2. **(B)** Percentage of biofilm remaining after treatment with different concentrations of *NaClO*. The blank control group was treated with DMSO. *NaClO* as a positive control. Data are presented as mean ± SD (*n* = 3, with three technical replicates). Statistical analysis was performed using Dunnett’s multiple-comparisons test, *p < 0.05.

The antimicrobial effects of ginsenosides are believed to be exerted through various mechanisms, including disrupting cellular structures, affecting metabolic pathways, inhibiting biofilm formation, and reducing biofilms (Wang et al., 2020). In the present study, the higher concentration of Rh2 required to reduce established biofilm biomass suggests that effective disruption of mature biofilms may involve both impairment of embedded bacterial cells and alteration of the extracellular matrix. Previous work has shown that Rh2 can penetrate and disrupt oral cariogenic biofilms (Cao et al., 2019), supporting the possibility that matrix disruption contributes to its activity against established biofilms. Nevertheless, because crystal violet staining primarily reflects total attached biomass, the reduction observed during biofilm formation cannot distinguish direct inhibition of biofilm development from a secondary effect resulting from inhibition of planktonic bacterial growth. Additional assays, such as viable-cell enumeration or normalization of biofilm biomass to bacterial growth, would be useful for defining the biofilm-specific effects of Rh2.

### 3.3 Rh2 reduces EPS-associated carbohydrate content in biofilms

EPS constitute a major component of bacterial biofilms and contribute to biofilm architecture, physicochemical properties, and antimicrobial tolerance (Li et al., 2021; Arias-Moliz et al., 2024; Ito and Ito, 2024). The extracellular matrix can modify the local chemical microenvironment and impede the penetration and activity of antimicrobial agents (Sharma et al., 2019). In the present study, Rh2 reduced EPS-associated carbohydrate content in a concentration-dependent manner over the range of 0.391–3.125 μg/mL. At the MIC, Rh2 reduced the measured EPS-associated carbohydrate content by 71.25% (*p* < 0.05), whereas *NaClO* produced a 20.25% reduction at its MIC (Figure 4). These findings are consistent with previous studies showing that Rh2 can inhibit extracellular polysaccharide production in cariogenic biofilms. Other ginsenosides have also been reported to affect the extracellular matrix of periodontal pathogens (Lei et al., 2025). However, the substantial reduction observed at the MIC should be interpreted in the context of the concomitant inhibition of bacterial growth. The phenol-sulfuric acid assay primarily measures carbohydrate-associated components of the biofilm matrix and cannot distinguish between reduced bacterial proliferation, impaired EPS production, and degradation or detachment of pre-existing matrix material. Therefore, the present findings support an association between Rh2 exposure and matrix depletion but do not by themselves establish direct inhibition of specific EPS biosynthetic pathways.

**Figure 4:**
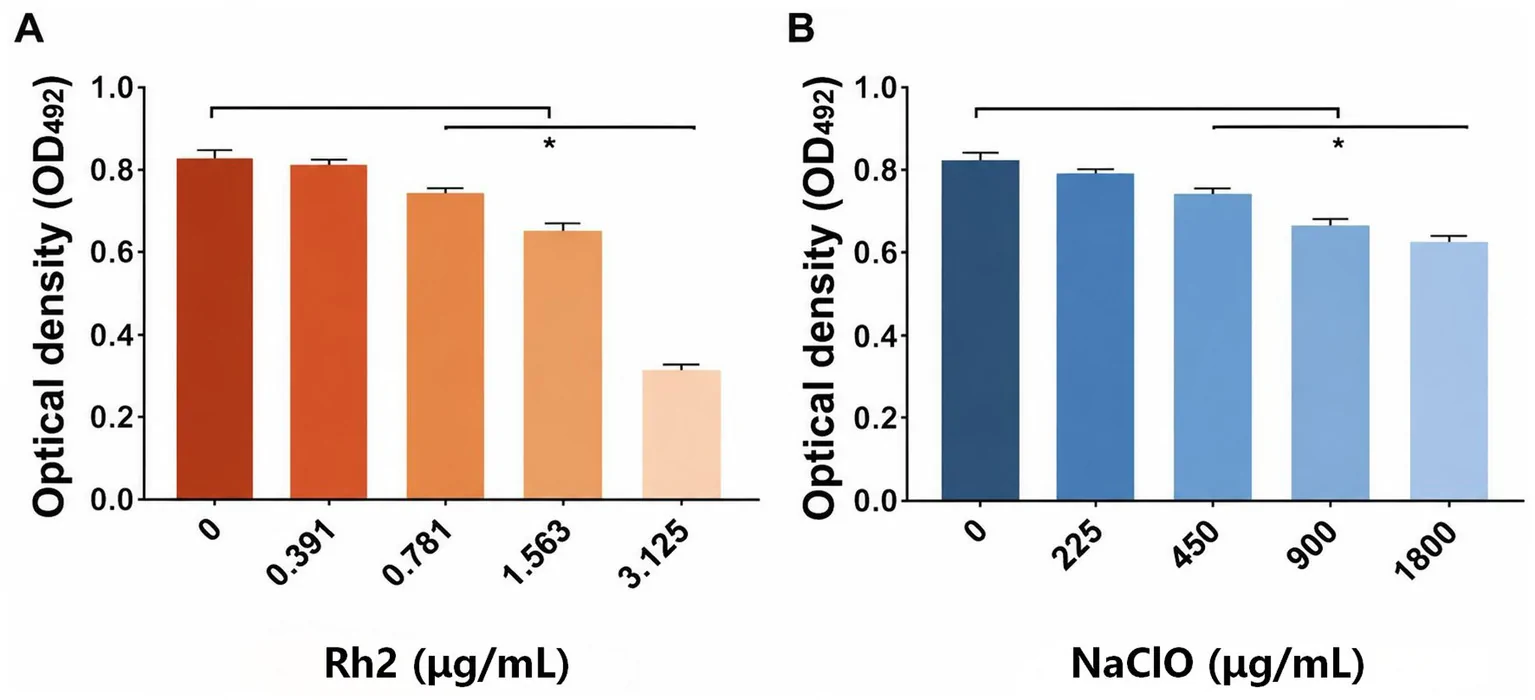
Ginsenoside Rh2 exhibits the ability to inhibit the synthesis of EPS in *E. faecalis* biofilms. **(A)** EPS synthesis in biofilms after treatment with different concentrations of Rh2. **(B)** EPS synthesis in biofilms after treatment with different concentrations of *NaClO*. The blank control group was treated with DMSO. *NaClO* as a positive control. Data are presented as mean ± SD (*n* = 3, with three technical replicates). Statistical analysis was performed using Dunnett’s multiple-comparisons test, *p < 0.05.

### 3.4 Rh2 reduces the metabolic activity of biofilms

The effect of Rh2 on the metabolic activity of *E. faecalis* biofilms was evaluated using the MTT assay. Rh2 reduced biofilm metabolic activity by 57.56 and 26.37% at 1 × MIC and 1/2 × MIC, respectively, compared with the control group (*p* < 0.05; Figure 5A). In contrast, 1/4 × MIC and 1/8 × MIC produced no significant change in metabolic activity. The reduction observed at 1/2 × MIC suggests that Rh2 may impair bacterial physiological activity before substantial loss of total biofilm biomass occurs. This finding is consistent with the concentration-dependent growth inhibition observed in planktonic cells and with the morphological and live/dead staining results described below. Collectively, these findings indicate that Rh2 affects not only biofilm biomass but also the metabolic state of bacteria within the biofilm. Because the MTT assay reflects cellular metabolic activity rather than directly quantifying viable cells, a reduction in MTT signal should not be interpreted as definitive evidence of bacterial death. Nevertheless, the concordance among the MTT, SEM, and CLSM findings supports an overall effect of Rh2 on the physiological integrity of biofilm-associated bacteria. At the MIC, *NaClO* produced a 21.34% reduction in metabolic activity (Figure 5B).

**Figure 5:**
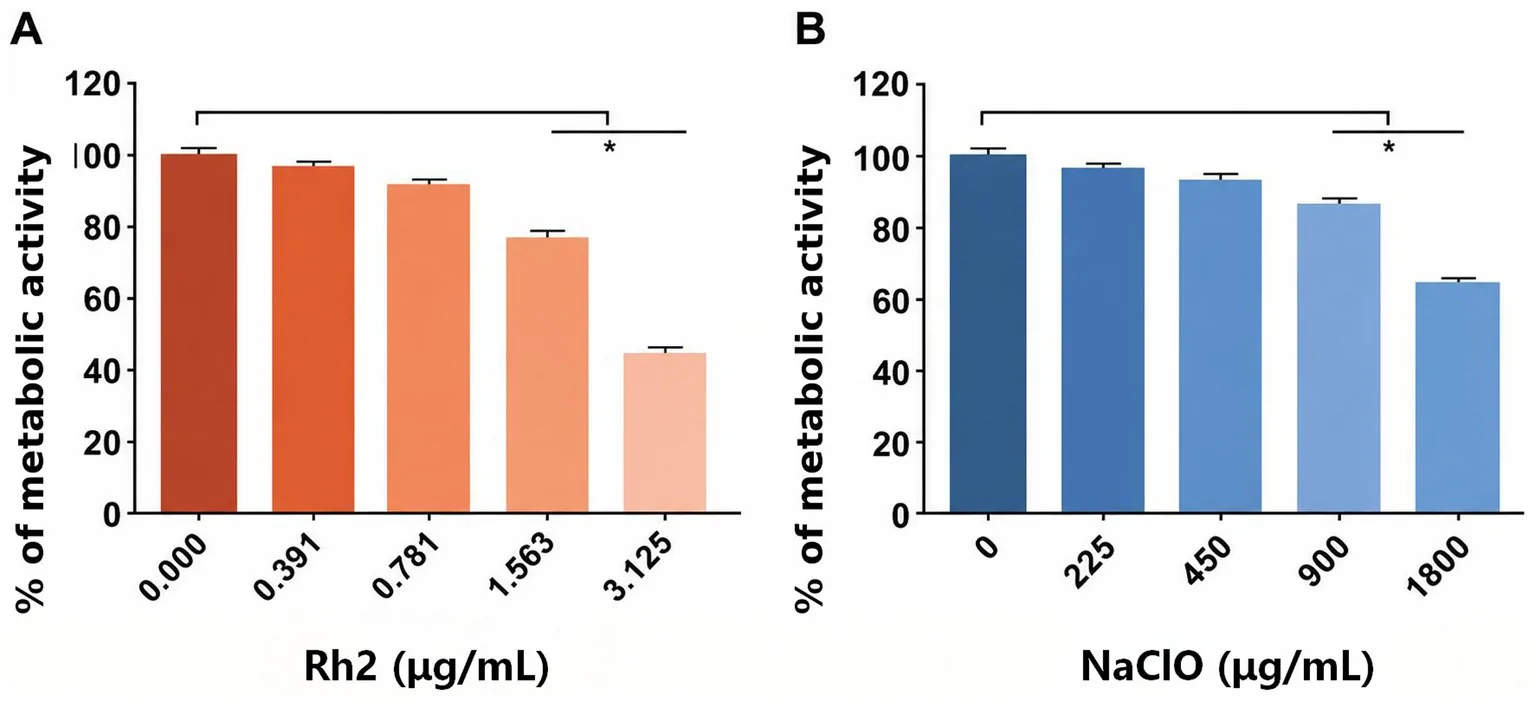
Ginsenoside Rh2 inhibits metabolic activity of *E. faecalis* biofilms. **(A)** Percentage of metabolic activity of biofilms by different concentrations of Rh2. **(B)** Percentage of metabolic activity of biofilms by different concentrations of *NaClO*. *NaClO* as a positive control. Data are presented as mean ± SD (*n* = 3, with three technical replicates). Statistical analysis was performed using Dunnett’s multiple-comparisons test, *p < 0.05.

### 3.5 Rh2 disrupts the structure of biofilms

SEM was used to examine the effects of Rh2 on the morphology and structural organization of *E. faecalis* biofilms. Compared with the control group, treatment with 1/2 × MIC Rh2 resulted in lower bacterial density, a less compact biofilm architecture, reduced apparent thickness, and roughened bacterial surfaces with visible pits (Figure 6). At 1/4 × MIC, the overall bacterial density was not markedly altered, although some cells exhibited roughened and concave surfaces. These morphological changes are consistent with previous evidence that Rh2 can interact with bacterial membranes and perturb membrane integrity. The structural alterations observed by SEM were accompanied by changes in live/dead fluorescence patterns, further supporting an effect of Rh2 on bacterial integrity within the biofilm. However, SEM provides morphological rather than direct functional evidence of membrane disruption; therefore, additional membrane-permeability and leakage assays would be required to confirm the underlying mechanism.

**Figure 6:**
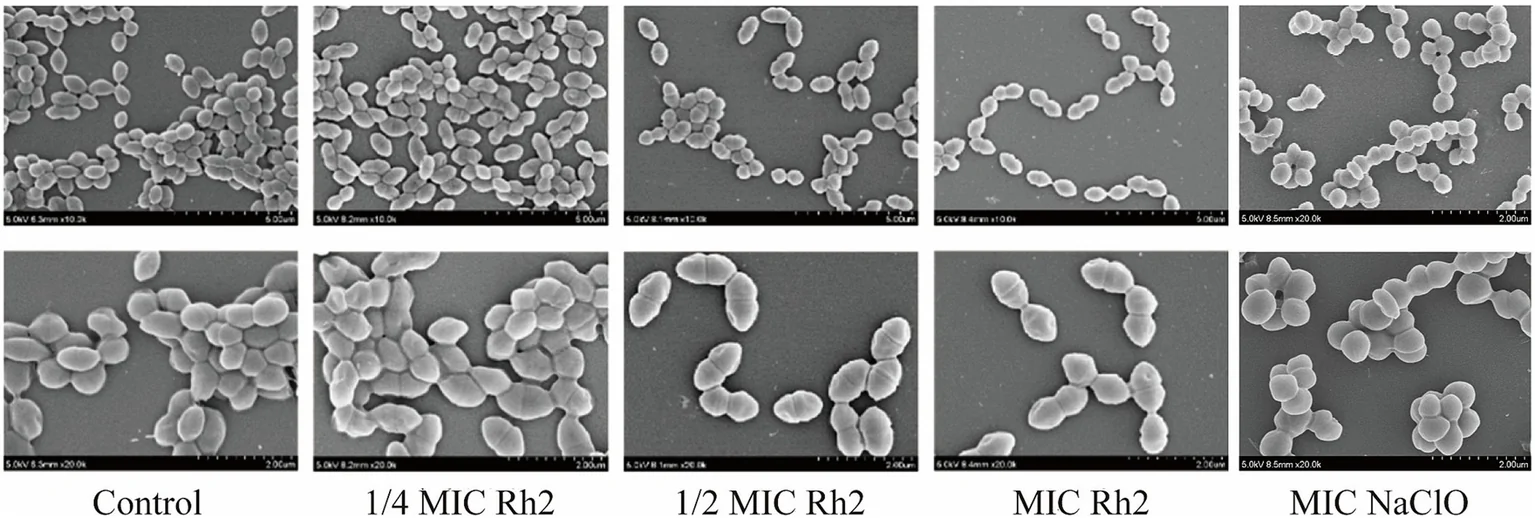
Representative scanning electron microscopy (SEM) images of *E. faecalis* biofilms. MIC, minimum inhibitory concentration. The blank control group was treated with DMSO. *NaClO* as a positive control. Three replicate samples were placed in each well.

### 3.6 Rh2 promotes cell death within the biofilm

CLSM was used to evaluate changes in bacterial membrane integrity within *E. faecalis* biofilms following Rh2 treatment. SYTO9 predominantly stains cells with intact membranes, whereas propidium iodide (PI) enters cells with compromised membranes and produces red fluorescence after binding to nucleic acids. In the control group, green fluorescence predominated, indicating a high proportion of cells with intact membranes. Following treatment with Rh2 at 1 × MIC and 1/2 × MIC, bacterial density decreased, green fluorescence was reduced, and red fluorescence became more prominent (Figure 7). Notably, at 1/4 × MIC, where little change in overall bacterial density was observed, green fluorescence still decreased whereas red fluorescence increased. These findings suggest that membrane damage may occur before a substantial reduction in biofilm biomass and may represent an early event in the antibacterial activity of Rh2. This interpretation is consistent with biophysical evidence showing that Rh2 interacts with membrane lipids and alters membrane permeability. However, because LIVE/DEAD staining primarily reflects membrane integrity, increased PI fluorescence should be interpreted as evidence of membrane compromise rather than definitive proof of irreversible cell death.

**Figure 7:**
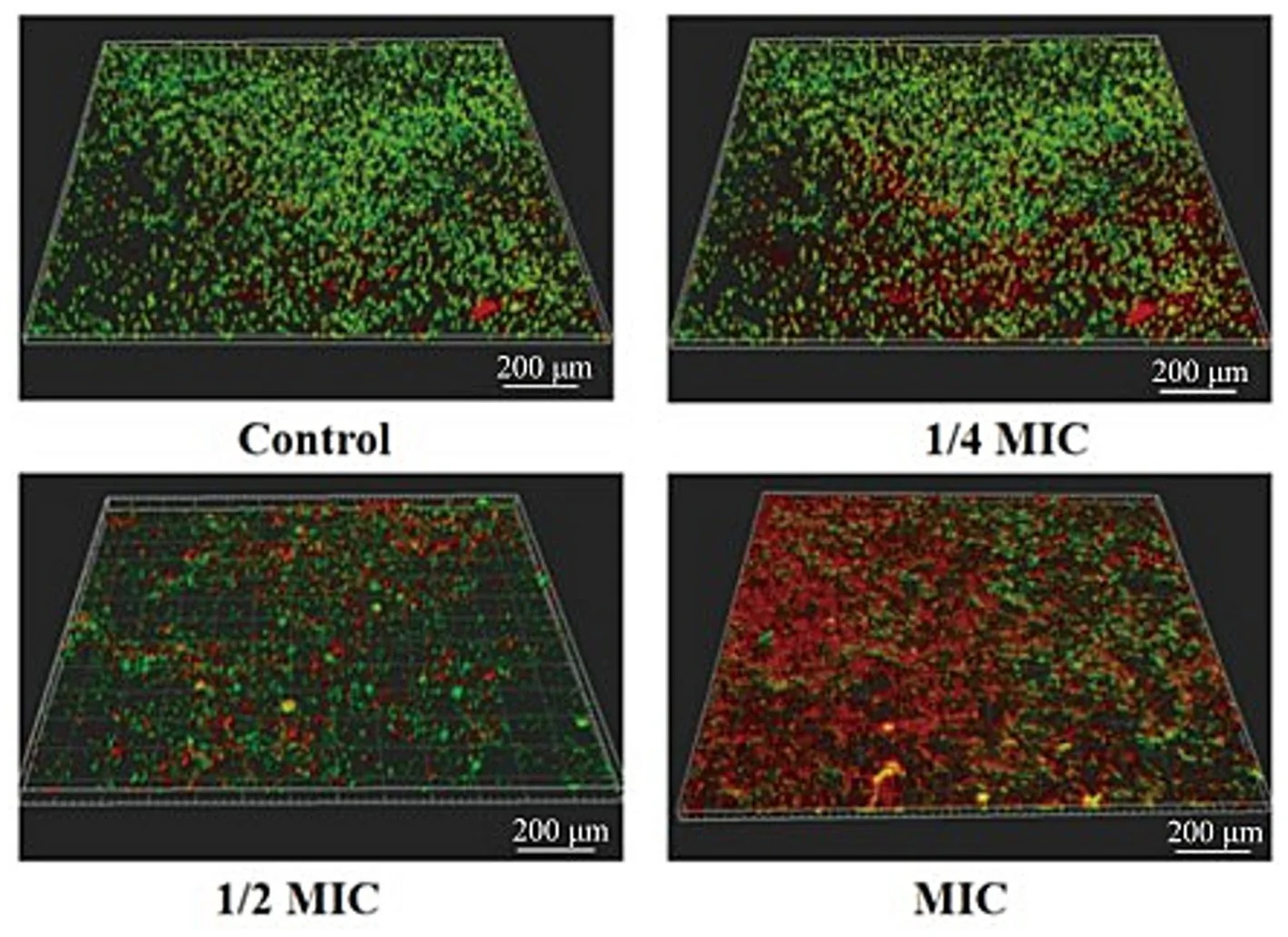
Representative 3D reconstruction of confocal laser scanning microscopy (CLSM) images of *E. faecalis* biofilms. MIC, minimum inhibitory concentration. The blank control group was treated with DMSO. *NaClO* as a positive control. Three replicate samples were placed in each well.

Taken together, the SEM and CLSM findings provide complementary morphological and functional evidence that Rh2 compromises the structural integrity of *E. faecalis* biofilms. These findings support a working model in which Rh2 interacts with the bacterial membrane, alters membrane integrity, and consequently disrupts intracellular homeostasis and metabolic activity (Chen et al., 2021). Membrane perturbation may also facilitate disruption of the surrounding extracellular matrix, thereby contributing to the loss of established biofilm structure. However, SEM and live/dead staining cannot determine the temporal sequence or molecular basis of these events. Further studies incorporating direct measurements of membrane permeability, intracellular leakage, ATP depletion, or membrane potential would therefore be required to establish the mechanism of action more conclusively.

### 3.7 Rh2 exhibits low cytotoxicity

Biocompatibility is an important clinical consideration for intracanal medicaments within the effective dose range, especially when the drug may extrude from the root canal and come into contact with periapical tissues, such as apical opening, root resorption, root foramen enlargement, and root perforation (Karobari et al., 2022). The cytotoxicity of Rh2 was assessed in L929 murine fibroblasts after 24 h of exposure using an MTT assay (Yang et al., 2020; Liu et al., 2023; Patil et al., 2023). This work demonstrated that the growth inhibitory effect of Rh2 on cells was dose-dependent (Figure 8A). Rh2 exhibited a concentration-dependent inhibitory effect on the survival rate of L929 cells, with an IC₅₀ value of approximately 20 μg/mL. Its MIC was 3.125 μg/mL, which is significantly lower than the concentration required to reduce the survival rate of L929 cells by 50%; this indicates that, under the experimental conditions, Rh2 exhibits a certain degree of *in vitro* selectivity in inhibiting bacterial growth. However, the difference between the cytotoxicity threshold and the concentration required to eliminate mature biofilms is small, particularly at 12.5 μg/mL. Therefore, the results of this study should be interpreted as indicating good cellular compatibility within the range of antibacterial concentrations tested, rather than as evidence of low overall cytotoxicity. *NaClO* at greater concentrations has considerable antibacterial properties against *E. faecalis*, but its cytotoxicity also rises with concentration. Rh2 demonstrated lower cytotoxicity against cultured cells than *NaClO*, indicating that it might be utilized as an intracanal medicament at low concentrations.

**Figure 8:**
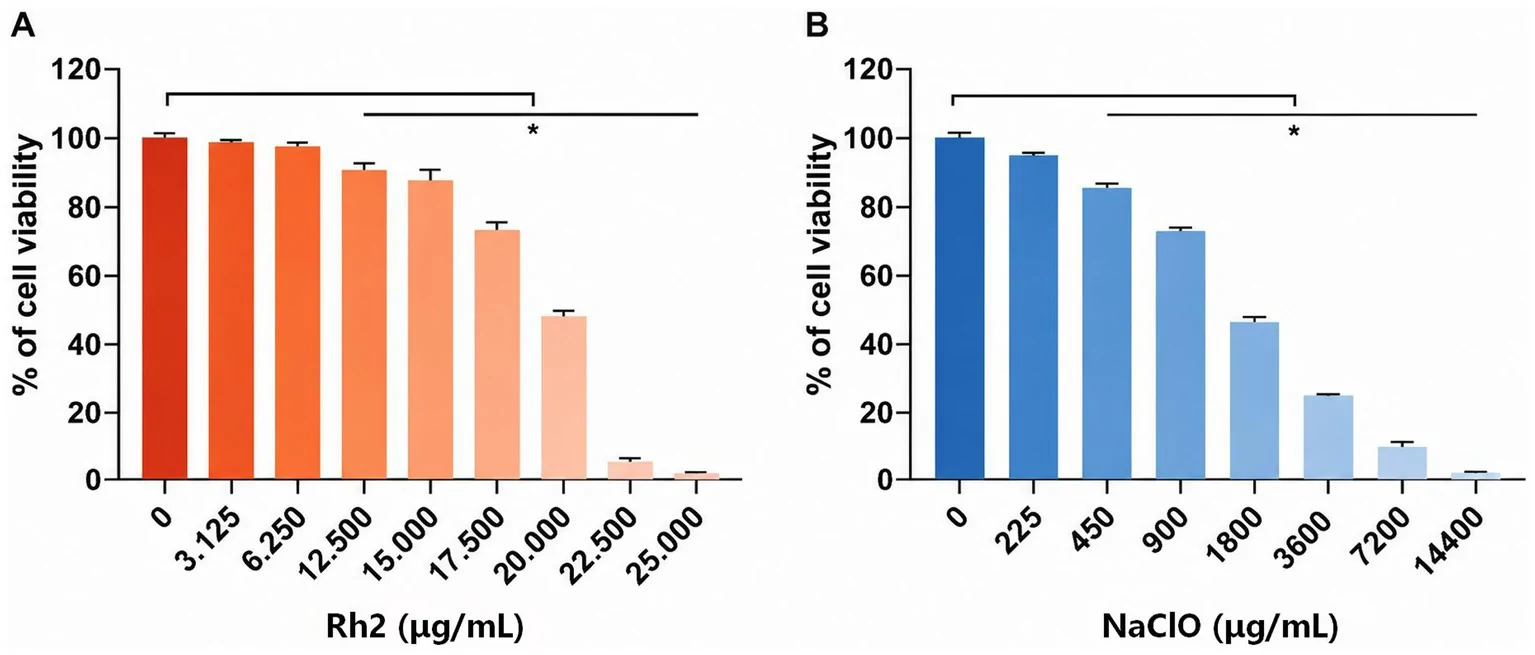
Cytotoxicity of ginsenoside Rh2 on L929 cells at different concentrations. The blank control group was treated with DMSO. **(A)** Percentage of cell viability of L929 by different concentrations of Rh2. **(B)** Percentage of cell viability of L929 by different concentrations of *NaClO*. *NaClO* as a positive control. Data are presented as mean ± SD (*n* = 3, with three technical replicates). Statistical analysis was performed using Dunnett’s multiple-comparisons test, *p < 0.05.

### 3.8 Limitations and future perspectives

First, the present study used only a single standardized strain, *E. faecalis* ATCC 29212. Although the use of a standardized reference strain improves experimental reproducibility and facilitates controlled comparisons among treatment groups, it may not capture the phenotypic and genetic heterogeneity of clinical *E. faecalis* isolates (Salem et al., 2022). Previous studies have demonstrated strain-dependent differences in biofilm formation, extracellular matrix production, virulence-associated characteristics, and responses to endodontic antimicrobial agents (Francisco et al., 2021; Manoil et al., 2023). Therefore, the antibacterial and antibiofilm effects of Rh2 observed in the present study should be further validated using multiple clinical isolates, particularly strains recovered from persistent or previously treated root canal infections.

Second, the present study employed a monospecies *E. faecalis* biofilm model. This approach is useful for evaluating the direct effects of Rh2 on a defined bacterial population and for minimizing interspecies variability; however, it does not fully reproduce the ecological complexity of root canal infections, which commonly involve polymicrobial communities (Parga et al., 2025). Interactions among bacterial species can influence biofilm architecture, metabolism, virulence, and antimicrobial tolerance. Recent studies using multispecies root canal biofilm models have also demonstrated that antimicrobial treatment can alter the composition and structure of the microbial community without completely eliminating the biofilm (Uttech et al., 2025). Although Rh2 has previously been evaluated against both monospecies and multispecies cariogenic biofilms, its activity against root canal-associated polymicrobial biofilms remains insufficiently characterized. Therefore, the antibiofilm effects observed in the present monospecies model may not fully predict the activity of Rh2 within complex root canal microbial communities.

Third, the biofilm experiments were conducted using conventional *in vitro* substrates rather than dentin or anatomically intact root canal models. Consequently, the present system does not reproduce the physical barriers and structural characteristics of the root canal environment, including dentinal tubules, isthmuses, irregular canal anatomy, and the resulting constraints on antimicrobial penetration and distribution (Tsesis et al., 2023). *E. faecalis* can penetrate deeply into dentinal tubules, and studies using extracted human teeth have demonstrated that conventional endodontic disinfectants may exhibit reduced efficacy against bacteria located within these structures (Mokhtari et al., 2024). Thus, although the present findings demonstrate the antibacterial and antibiofilm activity of Rh2 under controlled *in vitro* conditions, they do not establish whether Rh2 can adequately penetrate dentinal tubules, reach bacteria located in anatomically complex regions, or retain its antibacterial activity in an infected root canal. *Ex vivo* studies using extracted human teeth and anatomically relevant root canal models will therefore be important for assessing the translational potential of Rh2.

Furthermore, although the use of L929 fibroblasts provides a standardized and reproducible method for preliminary cell compatibility screening, there are several limitations to using a single cell type when assessing the biosafety of Rh2. In the context of root canal treatment, Rh2 may come into contact with periodontal ligament fibroblasts, gingival fibroblasts, osteoblasts, epithelial cells and immune cells, and these cell types may exhibit significant differences in their response to antimicrobial agents (Marcoux et al., 2020). Single-cell-type culture models are unable to adequately replicate the complex intercellular interactions and host responses that occur in periapical tissues. Antimicrobial agents may not only affect cell viability but also influence inflammatory signaling, immune cell function, extracellular matrix remodeling and tissue repair. Furthermore, traditional two-dimensional monolayer cultures lack the three-dimensional tissue architecture, extracellular matrix, diffusion barriers and multicellular interactions present *in vivo*, which may alter the local exposure of test compounds and the resulting biological responses (Cunha et al., 2024). Consequently, future studies should utilize clinically relevant human periodontal/periapical cell types, a variety of complementary cytotoxicity and inflammatory endpoints, and prioritise the use of multicellular or three-dimensional tissue models to evaluate Rh2. Such approaches will enable a more comprehensive assessment of the therapeutic window and enhance the translational relevance of the biosafety evaluation of Rh2.

## 4 Conclusion

This study demonstrated that 20(S)-ginsenoside Rh2 exerts antibacterial and antibiofilm effects against *E. faecalis* under controlled *in vitro* conditions. Rh2 inhibited planktonic bacterial growth and biofilm formation, reduced the biomass of established biofilms, decreased EPS-associated carbohydrate content and biofilm metabolic activity, and altered biofilm structure and membrane integrity. Rh2 also showed a relatively favorable cytocompatibility profile compared with *NaClO* within the tested conditions. These findings support further investigation of Rh2 as a potential intracanal antimicrobial compound. However, the clinical relevance of these findings remains to be established. Further studies using multiple clinical *E. faecalis* isolates, root canal-relevant polymicrobial biofilms, dentin-based and anatomically intact root canal models, and clinically relevant cell types are warranted to determine the efficacy, safety, and translational potential of Rh2.

## Data Availability

The original contributions presented in the study are included in the article/supplementary material, further inquiries can be directed to the corresponding authors.
